# ﻿*Gastrodiamicrochila* (Orchidaceae, Epidendroideae), a new species from Brunei Darussalam

**DOI:** 10.3897/phytokeys.256.149020

**Published:** 2025-05-02

**Authors:** Michal Hroneš, Michal Sochor, Alena Uvírová, Azlan bin Pandai, Salwana Jaafar, Rahayu S. Sukri, Martin Dančák

**Affiliations:** 1 Department of Botany, Palacký University, Šlechtitelů 27, CZ-77900 Olomouc, Czech Republic; 2 Centre of the Region Haná for Biotechnological and Agricultural Research, Czech Agrifood Research Center, Šlechtitelů 29, CZ-77900 Olomouc, Czech Republic; 3 Department of Ecology and Environmental Sciences, Palacký University, Šlechtitelů 27, CZ-77900 Olomouc, Czech Republic; 4 Brunei National Herbarium, Forestry Department, Ministry of Primary Resources and Tourism, Jln Menteri Besar, BB 3910, Bandar Seri Begawan, Brunei; 5 Institute for Biodiversity and Environmental Research, Universiti Brunei Darussalam, Jalan Tungku Link, BE 1410, Bandar Seri Begawan, Brunei

**Keywords:** Borneo, endemic species, holomycotrophic orchid, Malesia, mycoheterotrophy, tribe Gastrodieae

## Abstract

A new orchid species, *Gastrodiamicrochila* is described and illustrated. The species was found in a mixed dipterocarp forest in the Belait district, Brunei Darussalam, northern Borneo in 2024. It is distinct from most *Gastrodia* species in western Malesia by its small lip and column, and presence of the smooth cushion-like tissue on the inner side of lateral sepals. *Gastrodiamicrochila* is only the second species of *Gastrodia* recorded for Brunei Darussalam and the seventh known species for Borneo. A determination key for all *Gastrodia* species occurring in western Malesia is also provided.

## ﻿Introduction

The mycoheterotrophic flora of north-western Borneo (Sarawak and Brunei Darussalam) is exceptionally rich, including more than twenty species of *Thismia* (Thismiaceae), five species of *Epirixanthes* (Polygalaceae) and eleven species of *Sciaphila* (Triuridaceae) (Dančák et al. 2017; Dančák et al. 2020a, 2020b). The reported occurrence of only two species of *Gastrodia*, one from Sarawak and one from Brunei Darussalam (Wood et al. 2011; Hroneš et al. 2024), is thus in stark contrast with the diversity of other mycoheterotrophs and, more notably, with the six reported *Gastrodia* species from neighbouring Sabah (Wood et al. 2011). The more so, considering that *Gastrodia* is the second most species-rich genus of mycoheterotrophic plants globally (POWO 2024).

The genus, if treated in the wide sense, comprises 107 species (Khanal et al. 2024a, 2024b; POWO 2024; Qin et al. 2024; Chowlu et al. 2025) distributed from tropical western Africa and Madagascar, throughout southern and southeastern Asia, to Japan and eastern Siberia, eastern Australia, New Zealand and Pacific islands (POWO 2024). A comprehensive genus-wide taxonomic revision and phylogeny of *Gastrodia* is still lacking. The most recent infrageneric taxonomy proposed by Schlechter (1911), who recognised sections *Eu-Gastrodia*, *Codonanthus* and *Strogadia*, is not sufficient as it does not reflect known morphological diversity in the genus. However, an attempt to divide the genus into four separate genera *Demorchis*, *Leptogastrodia*, *Neoclemensia* and *Gastrodia* s. str. by Clements and Jones (2019) was also not widely accepted (Schuiteman 2021; POWO 2024). The rapidly increasing number of described species therefore requires a deeper focus on the evolution of the genus and the recognition of natural groups.

Species of *Gastrodia* are achlorophyllous, mycoheterotrophic herbs with reduced vegetative organs and fleshy tuberous rhizomes (Pridgeon et al. 2005; Ong 2015). Tubers of *G.elata* Blume are used in traditional medicine (Zhan et al. 2016). The flowers are resupinate to double resupinate, with three sepals and two petals connate into campanulate to tubular synsepalum inside which the lip and column are positioned (Pridgeon et al. 2005; Ong 2015). Many species are probably pollinated by small flies and display typical myophily pollination syndrome, i.e., white or brown-coloured flowers which emit a rotting fruit-like odour (Carr 1928; Martos et al. 2015). The flowers of some species are ephemeral, flowering for only a few days (Carr 1928, 1929). Due to their cryptic colours and short flowering period, many *Gastrodia* species are inconspicuous and easily overlooked in the field, which may explain why they are understudied in some areas such as Borneo (cf. Wong 2023).

In total, the occurrence of sixteen species is reported from western Malesia. Of these, only *Gastrodiajavanica* (Blume) Lindl. is widespread, *G.bambu* Metusala occurs in Java and Vietnam, *G.effusa* P.T. Ong & P. O’Byrne in Borneo and Malay Peninsula, *G.exilis* Hook.f. in southern India, Assam, Thailand, Cambodia and Sumatra, *G.spathulata* (Carr) J.J. Wood in Borneo and Java and *G.verrucosa* Blume in Java, Sumatra and Thailand (Ong and O’Byrne 2012; Metusala and Supriatna 2017; Suetsugu et al. 2017; Suetsugu et al. 2018a; POWO 2024). The remaining species are endemic to the respective island or peninsula, i.e. Borneo (3 species), Java (4 species), the Malay Peninsula (2 species) and Sumatra (1 species). In Borneo, the species diversity is concentrated in Sabah, with the presence of all six species reported from the island: *G.effusa*, *G.grandilabris* Carr, *G.javanica*, *G.maliauensis* Suetsugu, M. Suleiman & Tsukaya, *G.sabahensis* Wood & Lamb, and *G.spathulata* (Wood 2008; Wood et al. 2011; Suetsugu et al. 2018b). *Gastrodiajavanica* occurs also in Sarawak and *G.sabahensis* has recently been recorded from Brunei Darussalam (Wood et al. 2011; Hroneš et al. 2024). No species is known from Kalimantan. New discoveries are therefore likely.

During our ongoing research on mycoheterotrophic plants of Brunei Darussalam, we found a second species of *Gastrodia* for the country which did not resemble any species reported from Borneo. After a thorough examination of its morphology, it is described here as a new species.

## ﻿Material and methods

This study is based on the material collected in November 2024 in a forest on the northern slopes of Telingan Hill (Belait district), Brunei Darussalam. Morphological characters were studied on living plants and documented by macro photography. Morphological characters were compared with the protologues and relevant floristic literature of *Gastrodia* species from western Malesia, i.e. Borneo, Java, Peninsular Malaysia and Sumatra (Blume 1825, 1856; Smith 1903, 1921, 1931; Carr 1929, 1935; Wood 2008; Wood et al. 2011; Ong and O’Byrne 2012; Ong 2015; Tsukaya and Hidayat 2016; Metusala and Supriatna 2017; Suetsugu et al. 2018b; Schuiteman 2021). A voucher specimen has been deposited in BRUN (Thiers 2025). The morphological description was prepared from several individuals, but several floral characteristics (morphology of calli and column) are based on a single flower. Morphological description follows the terminology given in Ong (2015) and the level of information presented in Hroneš et al. (2024). The preliminary conservation assessments are based on the most recent version of the guidelines of the IUCN Standards and Petitions Committee (2024).

## ﻿Taxonomic treatment

### ﻿ Gastrodia microchila

Taxon classificationPlantaeAsparagalesOrchidaceae

Hroneš sp. nov.

722D5CE7-A142-5E85-B44C-4A5E1BC8E37C

urn:lsid:ipni.org:names:77360920-1

Figs 1 , 2 , 3

#### Type.

Brunei Darussalam • Belait district: Labi village, northern slopes of Bukit Telingan, mixed dipterocarp forest ca. 1.8 km E of primary school in Kampung Rampayoh, 4.37672°N, 114.47339°E, 200 m alt., 25 Nov 2024, *Hroneš 2024/4* (holotype: BRUN B 045 889).

#### Diagnosis.

*Gastrodiamicrochila* is similar to *G.holttumii* Carr from Peninsular Malaysia but differs by narrower cylindrical rhizome without distinct nodes (vs. robust, distinctly noded), flowers 8.5–9.0 mm wide, narrowly open (vs. 9.0–11.0 mm wide, widely open), free part of lateral and dorsal sepals oblong triangular to broadly triangular, up to 4.5 × 5.0 mm (vs. elliptic to oblong elliptic, 6.0–7.0 × 3.0–5.0 mm), free part of petals indistinct, ca. 0.7 × 0.6 mm, elliptic (vs. 1.0–2.0 × 0.7–1.0 mm, ovate), hypochile 2.0 × 2.0 mm, broadly oblong-orbicular (vs. 2.0 × 1.5 mm, oblong-ovate) and stelidia longer than anther cap (vs. subequal to anther cap).

**Figure 1.**
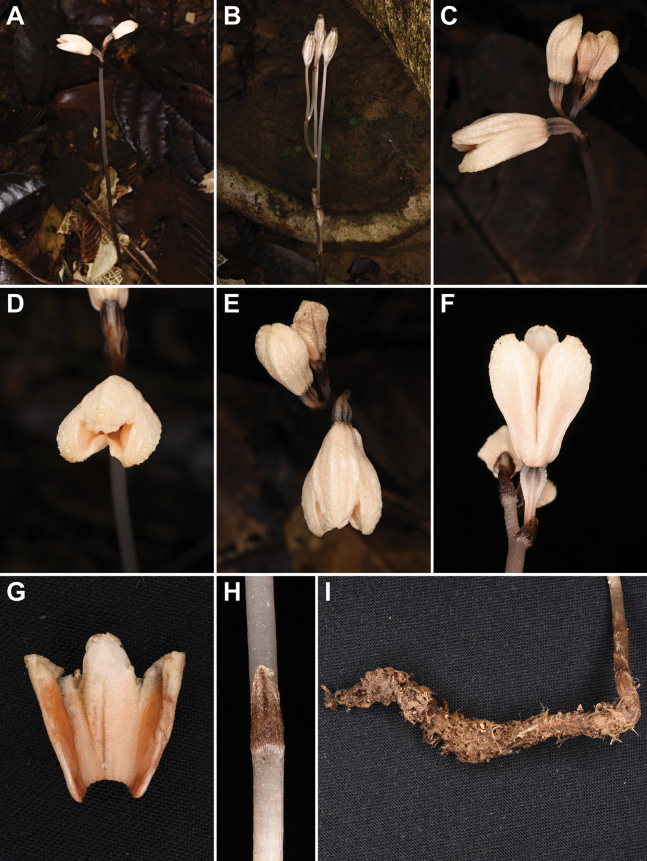
*Gastrodiamicrochila* Hroneš **A** habit of flowering plant **B** fruiting plant with capsules on elongated pedicels **C** detail of inflorescence with side view of flower **D** front view of flower **E** inflorescence viewed from above **F** flower viewed from below with connate and free part of lateral sepals, and ovary **G** dissected synsepalum showing minute petals and cushion-like smooth tissue on the internal side of sepals **H** part of the stem with leaf **I** tuber **A–I** from *Hroneš 2024/4* (Photo M. Hroneš).

#### Description.

Terrestrial, achlorophyllous herb, 16–21 cm tall. Rhizome ca. 50 mm long, 3–6 mm in diameter, tuberous, fleshy, cylindrical, without distinct nodes, densely covered by filiform scales. Stem 128.5–165.0 mm long, 1.8–2.2 mm in diameter, with 4–6 nodes, ± erect, indistinctly ridged, covered by low blunt verrucae, whitish brown, pale greyish brown to beige. Leaves 2.7–5.5 mm long, 2.5–3 mm wide at base, scale-like, broadly triangular, basally clasping, tip acute to erose, brown. Bracts subtenting base of each pedicel 2, unequal in size; longer one 4.0–4.5 mm long and 2.8–3.4 mm wide, broadly triangular, clasping basally, keeled, persistent, covered by low blunt verrucae, brown; shorter one 3.7–4.6 mm long, ca. 0.5 mm wide at base, 1.5–1.8 mm wide in the middle part, stipitate ovate to stipitate triangular with tapering tip, keeled, caducous in lower flowers. Inflorescence loose, 2–4 flowered; rhachis 8.5–10.5 mm long. Pedicel 2.5–5.3 mm long, 1.0–1.4 mm in diameter, elongating to 12.5–100.0 mm in fruit, with 6 distinct ribs, becoming smoother in fruit, greyish pink to pinkish orange. Flower (excluding ovary) 13.0–15.0 mm long, 8.5–9.0 mm wide, spreading almost horizontally, narrowly open, with sepals and petals basally fused, forming a five-lobed, tubular campanulate perianth tube; flower bud pinkish orange. Sepals externally pale pinkish orange, covered by low blunt verrucae (more distinct in young flowers), internally pinkish orange at base and whitish orange to beige apically. Lateral and dorsal sepals connate into synsepalum for ca. 2/3 of their length, lateral sepals connate for up to 1/2 of their length, connation perfect for basal 3–4 mm, additional 3–5 mm connate imperfectly, leaving a conspicuous furrow on the surface of flower; free portion 4.0–4.5 × 4.5–5.0 mm, oblong triangular, cucullate, blunt at apex, with irregular translucent margin, internally with beige-orange to deep orange-pink, cushion-like smooth tissue covering most of the sepal space. Free portion of dorsal sepal 5.0 × 5.0 mm, broadly triangular, blunt and shortly cucullate at apex, with irregular translucent margin. Free portion of petals indistinct, ca. 0.7 × 0.6 mm, elliptic, whitish orange to beige, almost translucent. Lip ca. 3.0 × 2.0 mm, adnate to base of column, greyish pink to beige; hypochile ca. 2.0 × 2.0 mm, thick and fleshy, almost flat, broadly oblong-orbicular; epichile 1.0 × 0.8 mm, oblong to almost rectangular with wide obtuse apex bent downwards, orange to reddish brown; keel ca. 0.4 mm wide, positioned at the transition of hypochile and epichile, hypochile part beige, epichile part reddish brown, with margins raised into two low ridges which have the tallest part at the transition between hypochile and epichile; calli ca. 0.2 × 0.2 mm, ± globose, pale orange-brown. Column ca. 3.0 × 1.9 mm, straight, oblong to rectangular, canaliculate, winged, without distinct rostellum, central part beige, wings slightly ventricose, beige, basally pale pinkish orange; stelidia ca. 0.8 mm long, terete, elliptic-triangular, obtuse, beige, slightly exceeding anther; anther cap ca. 0.6 × 0.9 mm, rectangular-ovate; pollinia not seen; stigma not seen. Ovary 2.3–5.0 × 1.8–2.6 mm, attached at an angle to pedicel, obconical, bluntly trilobed in intersection, slightly ventricose, with 3 high and 3 low ribs alternating to each other, covered by low blunt verrucae, greyish pink basally, greyish brown apically. Capsule 13.2–15.6 × 4.0–5.1 mm, cylindrical-fusiform, greyish pink in most part, brown apically.

**Figure 2.**
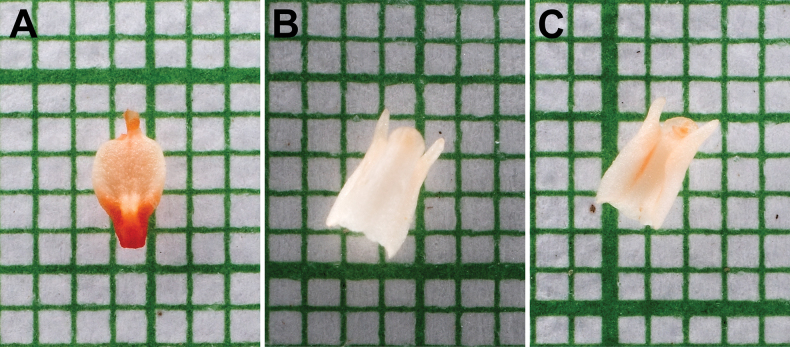
*Gastrodiamicrochila* Hroneš **A** lip **B** column, adaxial view **C** column, side-abaxial view **A–C** from *Hroneš 2024/4* (Photo M. Hroneš). The smallest grid = 1×1 mm.

#### Distribution and habitat.

Endemic to Brunei Darussalam. So far known only from its type locality in the Belait District. It grows in mixed dipterocarp forest on shady and humid slopes near the small streams. The forest was probably lightly logged in the past.

**Figure 3.**
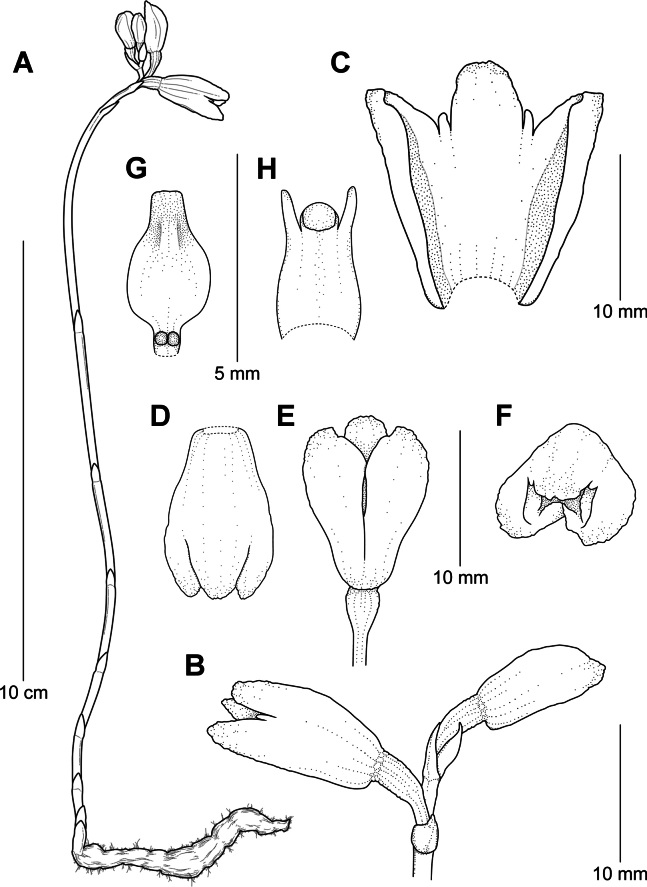
*Gastrodiamicrochila* Hroneš **A** habit of flowering plant **B** detail of inflorescence with bracts **C** dissected synsepalum with free parts of sepals and petals **D** flower viewed from above **E** flower viewed from below **F** front view of flower **G** lip **H** column **A–H** from *Hroneš 2024/4* (Illustration V. Blažek).

#### Etymology.

The name is derived from the Latin *micro*- = small and -*chilum* = lip, referring to the size of the lip, which is among the smallest in *Gastrodia* species in western Malesia.

#### Conservation status.

The species is currently known from a single small population spanning less than 500 m^2^. Fewer than ten individuals were observed at the site. The population is located in currently unprotected forest. Consequently, any random natural or human-induced event could destroy the site and the entire population. Therefore, we propose to preliminarily treat *G.microchila* as critically endangered (CR) based on the B2ab(iv)+D criteria.

## ﻿Discussion

The discovery of *G.microchila* represents the second known species of the genus in Brunei Darussalam (Hroneš et al. 2024). It is a valuable addition to the already rich mycoheterotrophic flora of northwestern Borneo and indicates that, despite the current progress, little is known about the actual diversity of these elusive plants.

The most (and only) similar species to *G.microchila* in western Malesia is *G.holttumii* from Peninsular Malaysia (Carr 1929; Ong 2015). Both species have lateral sepals internally with cushion-like smooth tissue covering most of the sepal space and very similar size and shape of lip and column but differ in dimension, shape and colour of several traits, especially the floral ones (see also Diagnosis). Unfortunately, the holotype of *G.holttumii* in SING contains only a fruiting plant, the flowers have not been preserved to the present (Ong 2015). Therefore, Ong (2015) selected a well-preserved flowering plant as an epitype which slightly differs in flower size and the shape of lip and column from the original description. Overall, *G.microchila* is more similar to the original description of *G.holttumii* (Carr 1929). *Gastrodiaholttumii* has relatively short and thick, “potato-like” tubers interrupted by very thin internodes, while *G.microchila* has long and relatively slender cylindrical tuber without distinct interruptions. The flowers of *G.holttumii* are 5–7 mm longer and ca. 2 mm wider than those of *G.microchila*. The colour of *G.holttumii* flowers differs between the original description and the epitype. It is described as pale yellow-brown (Carr 1929) or orange-brown with darker tips (Ong 2015), while it is pale pinkish orange in *G.microchila*. The lip colour in *G.microchila* is richer than in *G.holttumii*. Fruiting pedicel elongates in *G.holttumii* to 40 cm which is the longest in any *Gastrodia* from the Malay Peninsula (Carr 1929; Ong 2015). The pedicel elongation in *G.microchila* is quite variable, spanning 1–10 cm but does not reach the length observed in *G.holttumii*.

Due to its tubular-campanulate flowers covered by low blunt verrucae, *G.microchila* is also superficially similar to several *Gastrodia* species from western Malesia, especially *G.abscondita* J.J.Sm., *G.bambu*, *G.crispa* J.J.Sm., *G.maliauensis*, *G.selabintanensis* Tsukaya & A.Hidayat, and *G.verrucosa*. However, it differs from them by its very small lip and column which both are only ca. 3.0 mm long (vs. 4.0–12.0 mm long lip and 5.5–12.0 mm long column in the rest of the species). *Gastrodiamicrochila* also has very short petals when compared to other species. Its petals are less than 1 mm long, almost indistinct in some flowers. Another distinct morphological trait of *G.microchila* is the smooth cushion-like tissue located on the inner side of lateral sepals. To the best of our knowledge, this tissue is absent in the above-mentioned species, but we cannot rule out the possibility that it was overlooked, especially in species described from dry specimens (Hooker 1894; Carr 1929). This tissue is to some extent similar to verrucose tissue present in *G.callosa* J.J.Sm., *G.sabahensis* and *G.tembatensis* P.T.Ong & P.O’Byrne (Smith 1931; Wood 2008; Ong 2015; Hroneš et al. 2024) but differs from it by smooth surface.

### ﻿Key to the species of *Gastrodia* in western Malesia (Borneo, Java, Peninsular Malaysia, Singapore, and Sumatra)

**Table:** 

1	Perianth split between the lateral sepals almost to the base with lip visible between the lateral sepals, calli on the lip absent	***G.javanica***
–	Perianth tube completely connate basally for at least 1/3 of its length and enclosing the lip, calli on the lip present	**2**
2	Free part of petals inserted deep in synsepalum	**3**
–	Free part of petals terminal	**4**
3	Petals connate to synsepalum for 1.5 mm, apical part claviform, fimbriate	***G.spathulata***
–	Petals connate to synsepalum for 9.5 mm, apical part spatulate, glabrous	***G.dewildeorum***
4	Free part of petals almost of the same size and shape as sepals, lip and column protruding from the flower	***G.effusa***
–	Free part of petals distinctly smaller and of different shape as sepals, lip and column hidden inside the flower	**5**
5	Petals fimbriate, sepals smooth, fragile	***G.exilis***
–	Petals entire to irregularly dentate, sepals externally with low blunt verrucae, fleshy	**6**
6	Lateral sepals with verrucose cushion-like tissue on its inner surface	**7**
–	Lateral sepals with smooth cushion-like tissue or flat on its inner surface	**9**
7	Flowers (excl. ovary) ca. 11.8 mm long, ca. 9.0 mm in diam., lip ca. 5.0 mm long, hypochile 4.0 mm wide	***G.callosa***
–	Flowers (excl. ovary) 12.0–16.0 mm long, 13.0–28.0 mm in diam., lip 7.0–9.0 mm long, hypochile 4.5–6 mm wide	**8**
8	Flowers very widely campanulate, up to 28.0 mm wide, hypochile almost orbicular, column wings with claw-like thick process	***G.tembatensis***
–	Flowers campanulate, up to 12.5 mm wide, hypochile broadly elliptic, column wings with triangular process	***G.sabahensis***
9	Lip and column ca. 3.0 mm long, lateral sepals internally with cushion-like smooth tissue	**10**
–	Lip and column at least 4.0 mm long, lateral sepals without cushion-like tissue internally	**11**
10	Flowers 8.5–9.0 mm wide, narrowly open, free part of sepals up to 4.5 × 5.0 mm, oblong triangular to broadly triangular, free part of petals small, ca. 0.7 × 0.6 mm, stelidia longer than anther cap	***G.microchila***
–	Flowers 9.0–11.0 mm wide, widely open, free part of sepals 6.0–7.0 × 3.0–5.0 mm, elliptic to oblong elliptic, free part of petals distinct, 1.0–2.0 × 0.7–1.0 mm, stelidia subequal to anther cap	***G.holttumii***
11	Flowers relatively small, up to 10.0 mm long, free part of sepals up to 3.5 mm long, lip 4.0 mm long with hypochile ca. 2.7 mm wide	***G.verrucosa***
–	Flowers larger, more than 10.0 mm long, free part of sepals at least 4.0 mm long, lip at least 6.0 mm long with hypochile at least 3.5 mm wide	**12**
12	Lip white, yellowish white to brownish white	**13**
–	Lip brown, purplish brown to green	**15**
13	Flowers subglobose to widely campanulate, free part of sepals distinctly wider than long, lip broadly oblong to quadrate	***G.grandilabris***
–	Flowers tubular to tubular campanulate, free part of sepals of ca. same length and width, lip ovate to elliptic	**14**
14	Lip 7–8 mm long, shorter to about the same length as column	***G.crispa***
–	Lip 10–12 mm long, longer than column	***G.selabintanensis***
15	Flowers 17.0–20.0 × 14.0–16.0 mm in diam., lip 10.0–12.0 × 3.5–4.0 mm, oblong-lanceolate	***G.bambu***
–	Flowers up to 13.0 × 11.0 mm in diam., lip up to 7.0 × 4.0 mm, ovate-triangular to ovate	**16**
16	Petals 3.7 × 1.7 mm, oblong, lip green with orange apex	***G.abscondita***
–	Petals 1.8 × 2.2 mm, ovate to (sub)orbicular, lip dark brown with almost black apex	***G.maliauensis***

## Supplementary Material

XML Treatment for Gastrodia microchila

## ﻿Additional information

### Author ORCIDs

Michal Hroneš https://orcid.org/0000-0002-0380-929X

Michal Sochor https://orcid.org/0000-0002-6525-1295

Alena Uvírová https://orcid.org/0009-0000-8608-2032

Salwana Jaafar https://orcid.org/0000-0003-0224-9603

Rahayu S. Sukri https://orcid.org/0000-0002-2662-399X

Martin Dančák https://orcid.org/0000-0002-3851-4969

### Data availability

All of the data that support the findings of this study are available in the main text.
